# Energy use and the role of per capita income on carbon emissions in African countries

**DOI:** 10.1371/journal.pone.0259488

**Published:** 2021-11-04

**Authors:** Bosede Ngozi Adeleye, Romanus Osabohien, Adedoyin Isola Lawal, Tyrone De Alwis

**Affiliations:** 1 Department of Economics and Development Studies, Covenant University, Ota, Nigeria; 2 Regional Centre of Expertise (RCE) Ogun, Covenant University, Ota, Nigeria; 3 Centre for Economic Policy and Development Research (CEPDeR), Covenant University, Ota, Nigeria; 4 Department of Accounting and Finance, Landmark University, Omu-Aran, Nigeria; 5 Department of Economics, University of Colombo, Colombo, Sri Lanka; Shenzhen University, CHINA

## Abstract

This study contributes towards the realization of Sustainable Development Goal (SDG) 13 which aims *“take urgent action to combat climate change and its impacts”* by investigating the role of per capita income in moderating the impact of energy use on carbon emissions. Using data from 28 selected African countries covering 1990 to 2019 and deploying the FGLS, PCSE, and MM-QR techniques, findings reveal, among others, that: at the 1% significance level, a percentage change in energy use leads to between 0.60% and 0.70% increase in carbon emissions, on average, *ceteris paribus*. Correspondingly, income shows to be a positive driver of emissions contributing between 0.87% and 0.84% percentage increase, on average, *ceteris paribus*. Also, per capita income attenuates the impact of energy use on emissions by between -0.27% and -0.23%, on average, *ceteris paribus*. However, significant heterogeneities occur across the sub-regions. Specifically, Southern Africa shows the largest energy contributor to emissions 1.65% while Central Africa contributes the most to aggravating emissions by 1.87% through increase in per capita income. West Africa shows the largest moderation effect at -0.56%. Across the quartiles, the effects of energy use and per capita are positive. Given these, we submit that the strong correlation between energy usage and per capita income (i.e. economic growth) poses a dilemma for African economies in their drive for growth. Leaving room for trade-offs. Perhaps, the lesson is that as African countries seek for more development without contributing to carbon emissions, governments should invest more in renewable energy.

## 1 Introduction

Economic growth and development is hinged on activities involving the production, transportation and consumption of energy, it becomes nearly impossible for economic progress to occur without considerably impacting the environment via the emission of carbon dioxide. Therefore, given that climate change is mostly driven by carbon dioxide emissions [1,2], realizing Sustainable Development Goal (SDG) 13 which is to *“take urgent action to combat climate change and its impacts”* is a daunting task for developing economies like Africa. This is because the kind of energy used in Africa increasingly contributes to environmental problems [3,4]. The situation might likely worsen given the limited energy resource-base and increasing concentration on non-renewable energy sources like wood, coal, natural gas, and fossil fuels in the region. Aside that, the resulting environmental problems linked to energy use include: air pollution, solid waste disposal, water pollution, and thermal pollution, to mention a few. Hence, this study sets out to empirically contribute to the debate on measures to reducing the level of carbon emissions in Africa.

With per capita income as the proxy for economic growth, a review of some studies on the energy-growth-emissions dynamics in Africa shows that economic growth in the short-run significantly increases CO_2_ emissions [5]; energy use plays a significant role in increasing economic growth and financial development without aggravating pollution in Sub-Saharan Africa [6]; energy use has substantial and positive effect on economic growth [7,8]; biomass energy use lowers carbon emissions and a feedback effect between economic growth and carbon emissions exists [9,10]; economic growth exert a deteriorating impact on the environment in the short-run [11]; energy consumption and economic growth play key roles in environmental degradation and pollution in Africa [12]; energy intensifies carbon emissions [8,13]; economic growth aggravates CO_2_ emissions [14,15]; and that per capita energy has a significant long run effect in raising CO_2_ levels [16]. Given the interlocking impact of energy and economic growth on carbon emissions, it becomes necessary to examine if economic growth can stem the aggravating impact of energy on carbon emissions. Therefore, the role of per capita income in moderating the impact of energy use on carbon emissions which, to be best of knowledge, is yet to be empirically investigated gives the motivation for this study.

Having identified this research gap, this study complements the existing literature but differs distinctly by attempting to answer three questions: (1) does energy usage and income independently impact carbon emissions, (2) does the interaction of energy with income escalate or attenuate its impact on carbon emissions and (3) whether this impact significantly differs across the sub-regions? To tackle these questions, two empirical models are constructed. The first is a linear model that explores the direct relation between energy use, income and carbon emissions while the second model is a moderation model which is the inclusion of an interaction term into the first model. Further to addressing these questions, we make the following contributions to the literature: firstly, an unbalanced panel is engaged to allow for a sizeable number of African countries. After thorough scrutiny, 28 countries with sufficient data on the variables of interest: carbon emissions, energy use, and per capita GDP from 1990 to 2019 are selected. Secondly, that econometric defects may arise from panel data analysis if unchecked, this study applies the feasible generalized least squares (FGLS) and panel corrected standard errors (PCSE) techniques that control for cross-sectional dependence, autocorrelation and heteroscedasticity. Thirdly, in accounting for the possibility that the energy-income nexus may vary across the conditional distribution of the dependent variable, the method of moments quantile regression (MM-QR) developed by Machado and Santos [17] is deployed. Lastly, for informed comparativeness, the sample is divided into five sub-regional delineations–Central, East, North, Southern, and West Africa to observe if significant differences occur in the outcomes. The rest of the paper is structured as follows: section 2 reviews the extant literature; section 3 details the data and empirical approach; section 4 presents and discusses the results; and section 5 concludes.

## 2 Literature review

### 2.1 Carbon emissions and energy use

Without claiming to being exhaustive, a general overview on the link between carbon emissions, and the underlying variables–energy use, per capita income, and urbanization are discussed. Khan, Khan [18] investigates the existence of heterogeneous relationship among renewable energy, consumption, carbon dioxide emission and financial development by employing panel regression for trickling distributional and unobserved individual heterogeneity of the panel data set of 192 economies. The study notes that the variables are heterogeneous across quantile. In specific terms, the impact of renewable energy consumption on carbon emission is negative, increase in carbon emission leads to a fall in renewable energy, and improved financial development enhances renewable energy. The study highlights that no directional relationship flows from increase in both carbon emission and renewable energy consumption, and financial development. Jiao [19] examines the unidentified determinants of CO_2_ emissions in G7 economies and its impacts on renewable energy. The study employs second-generation panel co-integration techniques and observes that a stable long-run relationship exists among CO_2_ emission, trade, income, environmental innovation, and renewable energy consumption. Further findings reveal that policy instruments aimed at adjusting export, imports, income, and environmental innovation will have significant impact on CO_2_ emissions. The authors suggest employment of eco-friendly measure such as deployment of renewable energy techniques to achieve sustainable development.

Adams and Nsiah [20] employ a battery of econometrics techniques to examine the relationship among renewable energy, nonrenewable energy, economic growth and carbon emission for some selected 28 Sub-Sahara African economies from 1980 to 2014. Findings reveal that both renewable and nonrenewable energy sources induce carbon dioxide emission in the long-run, though the impact of renewable energy on carbon emission wanes in the short-run. Other outcomes are that a positive relationship exists between economic growth and environmental degradation while a negative association exist between urbanization and carbon emission such that 1% increase in GDP will induce a 1.3% and 1.82% increase in emissions in both the long- and short-run respectively.

Similarly, Ali, Abbas [21] reveal that increasing energy consumption causes a rise in carbon emissions in Lahore Metropolitan Area of Pakistan. Also, Hilfa, Mohamad [22] examine the nexus among CO_2_ emission and each of economic development, renewable energy, urbanization, and agriculture for the Malaysian economy based on data sourced from 1978 to 2016. The study employed the autoregressive distributed lag (ARDL) techniques and noted that a positive relationship exists between CO_2_ emission and each of economic growth and urbanization. The study also observed that though the impact of agriculture on emission is positive, the relationship is not significant in the long run. The study also noted that the relationship between CO_2_ emission and economic growth can best be described as an inverted-U suggesting the validity of the environmental Kuznets curve for the Malaysian economy. The study concluded that CO_2_ emissions has no direct bearing from modernization.

Similarly, Jin and Kim [23] examined the determinants of carbon emissions in selected 30 economies based on data sourced from 1990 to 2014. The study distinguished between the impact of renewable and nuclear energy uses and carbon emission by employing a several panel co-integration techniques as well as Granger causality tests. The results reveal that long-run relationship exist among carbon emission, renewable energy consumption and nuclear energy consumption. On the other hand, the result of the Granger causality test reveals that unlike renewable energy, nuclear energy has great impact on carbon emissions, suggesting that to reduce carbon emission and promote cleaner energy, concerted efforts should be put in place to enhance the expansion of the renewable energy sub-sector. The study concluded that renewable energy support growth and contributed significantly to reducing carbon emission, thus, ecological friendly.

### 2.2 Carbon emissions and income

Ivanovski, Churchill [24] analyzed the conditional convergence of per capita CO_2_ emission for 17 emerging market economy based on data sourced from 1921 to 2014. The results offer mixed outcomes for the presence of stochastic convergence for 11 out of 17 economies studied. The study also noted that the determinants of the observed behavior in relative per capita CO_2_ emissions reveal that income, population, financial development, and trade are the key drivers. Grunewald, Klasen [25] examined the theoretical connections between income inequality and per capita carbon dioxide emissions using robust panel data methods. The results show that higher income inequality is associated with lower carbon emissions for the low and middle-income economies, whereas higher income inequality increases per capita emissions for the upper middle-income and high-income economies. Liu, Jiang [26] employed a panel ARDL and quantile regression models to examine the impact of income inequality on carbon emissions across United States (US) both at long and short term. The study observed that higher income inequality increases US carbon emissions in the short-run with reverse reaction in the long-run. Bai, Feng [27] examined the link among income inequality, carbon emissions, renewable energy and technological innovation for the Chinese economic factoring sectional data from 2000 and 2015. The study employed a panel fixed effects regression techniques and a panel threshold model and observed that renewable energy technology significantly reduces per capita CO_2_ emissions; and that the impact is a single threshold induced, such that a lower threshold for income inequality will generate an insignificantly impact on emissions.

Furthermore, Streimikien, Sun [28] employed a partially linear model to examine the link among income inequality, economic activity and consumption-based greenhouse gas emissions for some selected economies based on data sourced from 1990 to 2014. The study noted that a rise in GDP per capita will induce significant upward movement in consumption-based carbon footprint per capita, up till a point where it begins to rise suggesting existence of a U-shaped model. The study also reported existence of several trends in relationship between inequality and carbon footprint per capita for the low-income inequality economies. The study noted that Veblen effect could be described as the motivating factor for fluctuations in the trends between inequality and carbon footprint per capita. Rojas-vallejos and Lastuka [29] examined the marginal effect of income inequality on carbon emission per capita for a panel of 68 economies based in a 50-year data sourced from 1961 to 2010. The study documented existence of a trade-off between carbon emissions and income inequality with the trade-off essentially being heterogeneous and depended on the level of development in each of these economies. It used a panel smooth transition regression model and noted that the relationship between income inequality and carbon emission is negative for economies with low moderate income per capita up till a point where it began to exhibit upward trends.

### 2.3 Carbon emissions and urbanization

Yao, Kou [30] employed the threshold regression model to examine the effect of urbanization process on carbon emissions for China based on data sourced from 30 Chinese provincial-level from 2001 to 2014. Three carbon emission measures employed are: carbon emission scale, per capita carbon emission, and carbon intensity. The study noted that urbanization can induce a downward trend in the three measures of carbon emissions, though the effect of the downward trend (abatement) reduces with a deepening urbanization. The study further observed that the impact of urbanization on carbon emission is mediated by four mediating variables such as technological progress, industrial structure, energy consumption structure and foreign direct investment.

Huo, Li [31] extended the work of Yao, Kou [30] by calibrating the role of urban buildings into the urbanization-carbon emissions nexus for China. The study examined the impact of urbanization on carbon emission from both quantity and structural dimensions, and categorized urbanization into three perspectives: population; economy and space. The results obtained revealed that a positive and significant relationship exist among urban population, the added value of tertiary industry and the urban building area and carbon emissions from a quantity dimension. The results of the structure dimension reveal that carbon emissions are essentially driven by urban population ratio and the proportion of tertiary industry on gross domestic product (GDP), though a negative relationship exist between the plot ratio of urban buildings and carbon emissions.

In another development, Liu and Liu [32] noted that technological limitation, wealth and population are the main drivers of carbon emissions in China, and that better urbanization exert a weaker effect on carbon emissions. The study also noted that carbon emissions demonstrate a strong spatial spillover impacts among different provinces in China. The study further observed that urbanization, technology, wealth and population sizes exert varying spatial interactive effects on carbon emissions in different part of China, thus, suggest that a one-for-all policy model may not work in reducing carbon emissions for China.

Han, Cao [33] noted that urban employment rate is the major driver of carbon emission intensity, with the per capita urban employment energy consumption having the least. Other factors of influence are urbanization rate, population intensity of GDP production and carbon emission density. This finding was obtained from analysis of the nexus between urbanization, carbon emissions, employment rate, population level based on based on data sourced from the Chinese economy from the years 2000 to 2018. The study employed suggestive econometric models to examine the relationship between these variables and recommends aggressive development of the urbanization maximizing the growth rate of GDP and minimizing petrochemical energy emissions, as key to achieving carbon emission control [34,35].

## 3 Variables, empirical model, and approach

### 3.1 Variables and expectations

To address the research questions, this study uses an unbalanced panel data from 28 selected African countries from 1990 to 2019. The selected countries are: Algeria, Angola, Benin, Botswana, Cameroon, Congo, Dem. Rep., Congo, Rep., Cote d’Ivoire, Egypt, Arab Rep., Ethiopia, Gabon, Ghana, Kenya, Libya, Mauritius, Morocco, Mozambique, Namibia, Niger, Nigeria, Senegal, South Africa, Sudan, Tanzania, Togo, Tunisia, Zambia, and Zimbabwe.

Country selection is based on availability of sufficient data on the key variables of interest–carbon emissions per capita (*EMS*), energy use per capita (*ENERGY*) and per capita GDP (*INCOME*). The control variable is urban population (*URBAN*). All four variables are sourced from World Bank (2020) World Development Indicators. *ENERGY* captures the rate at which the population consume energy. It is predictable that more energy usage either for personal or industrial use provokes more carbon emissions. Likewise, *INCOME* captures the average income of the population in a given year. It is anticipated that increase in income creates the demand for goods and services which fuels carbon emissions. In the same vein, the intensification of *URBAN* due to migration exerts pressure on infrastructural development which exacerbate carbon emissions. Given the expressed scenarios, positive coefficients are expected.

### 3.2 Econometric model

Similar to related studies [36–40], the variables are transformed to their natural logarithms and the study adopts a double log model to forestall the incidence of heteroscedasticity, outliers, and to establish elasticity relationships. The empirical model is set up by initially controlling for urbanisation after which financial development is included for robustness checks. The linear model which addresses the first question expresses carbon emissions as a function of energy usage and income level, shown in Eq (1):

$$\ln EMS_{it}=\alpha_{0}+\alpha_{1}\ln URBAN_{it}+\alpha_{2}\ln ENERGY_{it}+\alpha_{3}\ln INCOME_{it}+d_{t}+u_{it}$$ (1)

where, ln is natural logarithm; *EMS* is carbon emissions (metric tons per capita); *ENERGY* is energy use (kg of oil equivalent per capita); *INCOME* is GDP per capita (constant 2010 US$); *URBAN* is urban population (% of total population); *i* is the number of countries in the sample 1,2,…,*N*; *t* is the number of years 1,2,…,*T*; *d*_*t*_ captures the time trend included to control variations in the dependent variable; and *u*_*it*_ is the idiosyncratic error term that is independently and identically distributed (i.i.d).

To address the second question on whether *INCOME* moderates the impact of *ENERGY* on carbon emissions, Eq (1) is augmented to include the interaction term and the moderation model is expressed in Eq (2):

$$\ln EMS_{it}=\alpha_{0}+\alpha_{1}\ln URBAN_{it}+\alpha_{2}\ln ENERGY_{it}+\alpha_{3}\ln INCOME_{it}+\alpha_{4}\ln{\left(ENERGY*INCOME\right)}_{it}+d_{t}+u_{it}$$ (2)

Importantly, the sign of the coefficient of the interaction term, *α*_4_ gauges whether the interaction of income on energy usage heightens or curtails carbon emissions. A positive sign indicates that income intensifies the worsening impact of energy usage on carbon emissions and vice versa. Also, the statistical significance of *a*_4_ is relevant in the computation of the net effect of *ENERGY* on *EMS*. If it is statistically significant, then it is factored into the calculation of the net effect but if insignificant, it implies that it is statistically *not* different from zero and the net effect of *ENERGY* on *EMS* equates to its unconditional marginal effect. The net effect of energy usage on carbon emissions conditional on income is derived in Eq (3):

$$\frac{\partial \ln EMS}{\partial \ln ENERGY}=\alpha_{2}+\alpha_{4}\ln INCOME$$ (3)

Note, *α*_2_ is expected to be positive. So, if *α*_4_ > 0 it implies that income enables the worsening effect of energy use on carbon emissions. But if *α*_4_ < 0, the net effects of energy use will be contingent on the magnitude of the negative. Also, if the negative sign of *α*_4_ outweighs the positive sign of *α*_2_ then income improves the effects of energy usage on carbon emissions. On the other hand, if the negative sign of *α*_4_ is less than the positive sign of *α*_2_ it indicates that the ameliorating effect of income is not sufficient to constrain the positive weight of energy use on carbon emissions. Finally, if *α*_4_ = 0 it suggests that the interaction of energy use with income has no significant effect on carbon emissions.

### 3.3 Analytical approach

To logically expound the impact of energy and income on carbon emissions, the study adapts salient approaches explained chronologically. First, the analysis begins by observing the distinct properties of a variables (summary) and their correlated associations (correlated analysis). Secondly, the test for cross-sectional dependence (CSD) which is when the units in the panel are somewhat correlated in the model is carried out. Despite the obvious advantages of panel data analysis such as enhanced efficiency, greater degrees of freedom, and reduced incidence of multicollinearity among the variables, the problem of cross-sectional dependence may lead to wrong inferences if not checked [41–43]. The Pesaran [44] test for cross-sectional independence is carried out ascertain if there is CSD in the data. Thirdly, the outcome of the CSD test determines whether to engage the first- or second-generation unit root tests. In the event of CSD in the model, the second-generation Pesaran [44] augmented Dickey-Fuller unit root test suited for cross-sectionally dependent panels is performed. Otherwise, the Levin, Lin [45] unit root test suffices. Fourthly, to determine if the variables are cointegrated, that is, if a long-run association exists, the choice is made between invoking the Westerlund [46] cointegration test which is a second-generation test or the Kao [47] cointegration test in the event of cross-sectional independence.

Lastly, Eqs (1) and (2) are analysed using the panel-corrected standard errors (PCSE) and feasible generalized least squares (FGLS) techniques. These procedures which have been used by related studies [7,48–50] control for cross-sectional dependence, autocorrelation, and heteroscedasticity. These methods fit a linear cross-sectional time-series model on the assumption that the errors are by default heteroscedastic and contemporaneously correlated across panels. These estimators also correct the standard errors of the coefficient estimates for possible dependence [51,52]. Using the PCSE and FGLS techniques is to serve as robustness for one another so as to observe the consistency of the impact of energy and income on carbon emissions.

For more robustness checks and given that the PCSE and FGLS techniques deal only with the conditional mean of *EMS*, this study controls for distributional heterogeneity using novel method of moments quantile regression (MM-QR). The approach which is developed by Machado and Santos [17] is robust for handling fixed effects in panel quantile models and allows for the estimation of other aspects of the conditional distribution (25^th^, 50^th^, and 75^th^ quantiles) of the dependent variable. Modifying Eq (1) and following Machado and Santos [17], Anser, Adeleye [53], and Ike, Ojonugwa [54], the conditional quantile *Q*_*EMS*_(*τ*|*X*_*it*_) estimation of the location-scale variant model takes the following general specification, shown in Eq (4):

$$Q_{EMS}(\tau |X_{it})=(\alpha_{i}+\delta_{i}qi)+X_{it}^{'}\beta +Z_{it}^{'}\gamma q(\tau )$$ (4)

Where, Eq (4) is assumed to be a linear model; $X_{it}^{'}$ is a vector of all explanatory variables used in the study; *Q*_*EMS*_(*τ*|*X*_*it*_) represents the quantile distribution of the dependent variable conditional on the location of explanatory variables; *α*_*i*_(*τ*) = *α*_*i*_*+* *δ*_i_*q*(*τ*) is the scalar coefficient of the quantile-*τ* fixed effect for individual *i*, or the distributional effect at τ; ***Z’*** is a *k*-vector of known differentiable (with probability 1) transformations of the components of ***X*** with element *l* where *l* = 1,…,*k*; *q*(*τ*) is the *τ*-th quantile derived from the following optimization function:

$$\underset{q}{\min}\sum_{i}\sum_{t}\rho_{\tau }({\hat{R}}_{it}-({\hat{\delta }}_{i}+Z_{it}^{'}\hat{\gamma })q)$$ (5)

Such that, *ρ*_*τ*_ (*A*) = (*τ* - 1)*AI*{*A* ≤ 0}+*τAI*({*A* > 0}) represents the check-function.

## 4 Analysis and interpretations

### 4.1 Correlation analysis and summary statistics

The association between the regressors and the dependent variable vis-à-vis the associations among the explanatory variables are shown in the upper panel of Table 1. All the regressors show positive and statistically significant relations at the 1% level with carbon emissions. Similarly, relations among the regressors are positive and statistically significant the 1% level. At a glance, some concern may arise from the 0.771 correlation coefficient between *URBAN* (a control variable) and *INCOME* (predictor variable) but the variance inflation factor (VIF) of 1.87 shown in the lower panel of Table 2 gives the assurance that the model does not suffer from the problem of multicollinearity.

**Table 1:** Correlation analysis and summary statistics.

Variable	*EMS*	*URBAN*	*ENERGY*	*INCOME*
*EMS*	1.000			
*URBAN*	0.743***	1.000		
*ENERGY*	0.795***	0.54***	1.000	
*INCOME*	0.908***	0.771***	0.718***	1.000
*Descriptive Statistics*				
*Full Sample*: Observations	754	810	687	831
Mean	1.501	43.895	741.715	2647.444
Standard Deviation	2.258	16.817	662.709	2664.432
Minimum	0.008	12.621	113.091	164.337
Maximum	9.998	89.741	3353.528	12064.781
*Central Africa*: Observations	135	150	125	150
Mean	1.044	56.313	671.455	3434.848
Standard Deviation	1.365	15.812	662.836	3473.797
Minimum	0.008	30.633	226.984	276.056
Maximum	4.919	89.741	3129.079	11949.282
*East Africa*: Observations	189	210	173	210
Mean	0.614	29.601	582.508	1650.87
Standard Deviation	0.86	9.024	192.483	2295.84
Minimum	0.041	12.621	361.166	164.337
Maximum	3.442	44.072	1111.422	10949.243
*North Africa*: Observations	160	180	150	171
Mean	2.984	56.068	1024.632	3443.375
Standard Deviation	2.769	15.141	862.687	2427.899
Minimum	0.107	28.61	307.104	754.837
Maximum	9.998	80.393	3353.528	12064.781
*Southern Africa*: Observations	81	60	74	90
Mean	4.052	47.233	1433.417	5765.332
Standard Deviation	3.4	12.555	845.947	1291.997
Minimum	0.028	27.656	470.464	3501.271
Maximum	9.979	70.172	2950.154	8092.965
*West Africa*: Observations	189	210	165	210
Mean	0.365	37.93	394.453	1097.232
Standard Deviation	0.185	10.565	174.348	501.527
Minimum	0.049	15.368	113.091	426.684
Maximum	0.809	56.707	798.63	2563.9

Note: ***, **, and * represent statistical significance at the 1%, 5%, and 10% levels, respectively; EMS = Carbon Emissions.

Source: Authors’ Computations.

**Table 2:** Full sample results (Dep. Variable: lnEMS).

*Variables*	*FGLS (Main)*		*PCSE (Robustness)*	
	[1]	[2]	[3]	[4]
ln*URBAN*	0.361***	0.304***	0.278*	0.200
	(4.542)	(3.273)	(1.908)	(1.226)
ln*ENERGY*	0.598***	2.688***	0.695***	2.414***
	(14.20)	(8.046)	(9.678)	(5.584)
ln*INCOME*	0.865***	2.568***	0.841***	2.236***
	(29.99)	(10.20)	(21.95)	(7.269)
lnENERGY*INCOME		-0.270***		-0.225***
		(-6.875)		(-4.760)
*Net Effects*		0.69%		0.75%
*CONSTANT*	-87.76**	-107.7**	0.0000	0.0000
	(-2.016)	(-2.522)	(.)	(.)
Variance Inflation Factor (VIF)	1.87		1.87	
No. of Obs./Groups	653/27	653/27	653/27	653/27
R-Squared			0.848	0.716
Wald Statistic	6606***	2735***	5934***	3534***
Time Dummies	Yes	Yes	Yes	Yes

Note: ***, **, and * represent statistical significance at the 1%, 5%, and 10% levels, respectively; z-statistics in (); EMS = Carbon Emissions.

Source: Authors’ Computations.

The lower part of Table 1 details the historical properties of the variables but discussions are limited to the three variables of interest which are carbon emissions, energy use, and income. The average emissions per capita for the full sample is 1.501 and the standard deviation of 2.257 shows a closer distribution around the sample mean. Among the regions, Southern Africa region shows to have the highest average value of 4.052 emissions per capita Cursory look at the data reveals that Libya (North Africa) has the highest emissions per capita of 9.998 in 2010 followed by South Africa (Southern Africa) with 9.979, 9.967, 9.519, and 9.499 in 2008, 2009, 2004, and 2007, respectively. These figures also confirm Libya and South Africa being listed among the four African countries with emissions per capita higher than the world average of 1.3 metric tons per year. The other two countries are Seychelles and Equatorial Guinea. Correspondingly, report from Carbon Brief details South Africa as the 14^th^ largest emitter of greenhouse gases (GHGs) in the world. The sample’s mean value for energy use per capita is 741.7 and across the regions Southern Africa has the highest (1433.42) followed by North Africa (1024.63). From the data, Libya consistently ranks 9 out 10 highest energy users by countries from 2995.87 energy use per capita in 2001 to 3353.53 in 2010. Gabon (East Africa) also ranks fifth in the data with 3129.08 value in 2010. On per capita income, the average value for the full sample is US$2,647.44 and the standard deviation of 2664.43 indicates a wide dispersion from the sample average. Across the regions, Southern Africa ranks highest with the average value of US$5,765.33 followed by North and East Africa with US$3,443.38 and US$3,434.85, respectively. Ranking the countries, Libya (North Africa) and Gabon (Central Africa) ranks in the top 10. For instance, Libya is highest with per capita income of US$12,064.78 in 2010 followed by Gabon with US$11,949.28 in 1998.

### 4.2 Pre-estimation statistics

Prior to engaging the econometric analysis, the test for cross-sectional dependence (CSD), unit root and cointegration are performed. The pre-estimation results shown in Table 3 reveal the presence of CSD at the 1% significance level. The series also show mixed level of integration from the CADF results with carbon emissions, energy use and income at first difference stationarity while urbanisation and financial development at level stationary. Lastly, Westerlund (2007) cointegration results reject the null hypothesis of no cointegration with and without cross-sectional means at the 1% significance level.

**Table 3:** CSD, panel unit root, and cointegration tests.

Variable	*EMS*	*URBAN*	*ENERGY*	*INCOME*
Pesaran (2007) CD-test	25.401***	68.537***	25.977***	51.361***
*Pesaran Cross-sectional ADF Unit Root Tests*				
Level	-1.485	-2.179**	1.095	-0.054
1st Difference	-12.587***	N/A	-8.425***	-7.564***
*Westerlund Cointegration Test*				
With cross-sectional means	-3.9996***			
Without cross-sectional means	-2.8448***			

Note: ***, ** represent statistical significance at the 1% and 5% levels, respectively; EMS = Carbon emissions; N/A = Not applicable.

Source: Authors’ Computations.

### 4.3 FGLS and PCSE results—full sample

Columns [1], [3] and [2], [4] relate to Eqs (1) and (2), respectively using the FGLS and PCSE approaches. On the impact of energy use and per capita income on carbon emissions while controlling for urbanization, findings show that a percentage change in energy leads to between 0.598 and 0.695 percentage increase in carbon emissions, on average, *ceteris paribus*. This relationship is statistically significant at the 1% level. Correspondingly, income shows to be a positive driver of emissions contributing between 0.865 and 0.842 percentage increase, on average, *ceteris paribus*. Comparatively, the income elasticity is higher than that of energy elasticity which gives the indication that income is the more dogged driver of carbon emissions. On energy and income as positive predictors of emissions, our findings align with previous studies [7,14,21,55–63]. This positive relation is not far-fetched. One, non-renewable energy is divided into four components: coal, natural gas, oil, and nuclear energy. These energy variants are used regularly by individuals and corporations evidencing an active and vibrant economy with the resultant effect of contributing to carbon emissions. Second, a growing economy evidenced by increase in per capita income creates the urge to demand for local and foreign goods by the people which is usually to gratify flamboyant lifestyles. This demand is satisfied by the absorptive capacities of the industrial sector who use non-renewable energy during the production process. Consistent with previous findings [64–69] urbanisation significantly contributes to carbon emissions at the 1% and 10% levels, respectively. Influx into urban areas puts pressure on the quest for goods and services which stimulates aggregate demand via production permitting the emission of carbon dioxide. Finally, a cursory look at columns [1] and [3] reveal that the estimated elasticity for per capita income represents the highest implying that an increase has a greater impact on carbon emissions in Africa, followed by energy usage, and urbanization.

On whether per capita income moderates the effect of energy use on carbon emissions, columns [2] and [4] reveal that the coefficients of the interaction term are negative and statistically significant at the 1% level. This finding is novel to the literature. To this end, with the influence of income, the net effect of energy on carbon emissions is computed as: 0.69% (that is, 2.688 - [0.270*$\bar{\ln INCOME}$) for the FGLS model and 0.75% by similar computation for the PCSE model. Thus, it is plausible to argue that the positive interaction of per capita income and energy leads to a 0.69% and 0.75% increase in emissions, on average, *ceteris paribus*. Though both energy use and income individually intensify emissions, their intrinsic combination decelerates it. The most plausible reasoning is that with increased income, the urge to use more environmentally friendly energy sources may arise. That is, individuals and corporations may shift from using non-renewable depletive energy like firewood, coal, crude oil, natural gas and nuclear energy to renewable energy sources like solar, wind, geothermal, and hydro-energy. With the interaction term, urbanization shows to have a statistically significant effect on carbon emissions, howbeit for the GLS model only. Having controlled for time dummies, the models diagnostics reveal that the variance inflation factor of 1.87 shows that there is no multicollinearity problem. The R-squared reveal that between 72% and 85% variation on carbon emissions are explained by the regressors while the Wald statistics provides evidence that all the variables are jointly significant in predicting carbon emission.

### 4.4 FGLS and PCSE results–regions

Tables 4 and 5 show the results from the FGLS (main analysis) and PCSE (robustness checks) techniques which will be interpreted simultaneously to avoid repetitions. For each region, the first and second columns relate to the first and second study questions. Inferences from the first columns indicate that with the exception of Central Africa, other regions exhibit a statistically significant positive energy-emissions relation at the 1% level. The region with the highest energy elasticity is Southern Africa with 1.652 followed by East Africa with 1.309 suggesting that elastic relationships hold such that percentage change in energy use increases carbon emissions by more than a percentage increase in both regions. With particular reference to Southern Africa, this magnitude gives some empirical backing to various reports [1,2,70,71] suggesting that Southern Africa is one of the world’s highest contributor to carbon emissions. On the impact of income, all the regions with the exception of Southern Africa exhibit statistically significant relation with carbon emissions at the 1% and 5% levels, respectively amongst which Central Africa has the highest income elasticity with 1.740. This also implies an elastic relationship. In essence, emissions are aggressively driven by income status in Central Africa. Urbanization slows emissions in Central and Southern Africa and intensifies in East, North, and West Africa. These relationships are statistically significant at the 1% level. The plausible argument is that higher population density, and larger settlements which are features of urbanization stimulate consumption (demand) which in turn induces production that could pose a threat to green and clean environment via increase in carbon emissions [72–74].

**Table 4:** FGLS results for the regions (Dep. Variable: lnEMS).

*Variables*	*Central Africa*		*East Africa*		*North Africa*		*South Africa*		*West Africa*	
	[5]	[6]	[7]	[8]	[9]	[10]	[11]	[12]	[13]	[14]
ln*URBAN*	-1.376*	-1.028	0.832***	0.630***	1.116***	1.897***	-0.550*	-0.583*	0.854***	0.314
	(-1.921)	(-1.210)	(3.768)	(3.011)	(3.231)	(5.637)	(-1.902)	(-1.875)	(5.029)	(1.214)
ln*ENERGY*	-0.0860	1.196	1.309***	5.209***	0.698***	1.415**	1.652***	2.194	0.564***	4.287***
	(-0.570)	(0.734)	(5.470)	(4.883)	(8.681)	(2.027)	(9.119)	(1.248)	(5.950)	(2.976)
ln*INCOME*	1.740***	2.461***	0.632***	3.464***	0.121**	0.897	0.462	0.991	0.322***	3.733***
	(11.94)	(2.664)	(7.874)	(4.448)	(2.246)	(1.363)	(1.464)	(0.571)	(2.887)	(2.800)
lnENERGY*INCOME		-0.140		-0.455***		-0.0945		-0.0651		-0.540**
		(-0.788)		(-3.698)		(-1.147)		(-0.311)		(-2.570)
*Net Effects*		0.00		2.10%		1.42%		0.00		0.56%
*CONSTANT*	-140.6	-144.7	0	0	-85.40*	-112.9**	-348.2***	-351.9***	-32.94	-52.00
	(-0.601)	(-0.618)			(-1.799)	(-2.249)	(-3.930)	(-3.947)	(-0.383)	(-0.597)
No. of Obs./Groups	125/5	125/5	173/7	173/7	141/6	141/6	49/2	49/2	165/7	165/7
Wald Stat	1233.52***	1223.30***	1751.32***	2880.86***	1771.62***	918.19***	934.49***	935.32***	271.86***	890.49***
Time Dummies	Yes	Yes	Yes	Yes	Yes	Yes	Yes	Yes	Yes	Yes

Note: ***, **, and * represent statistical significance at the 1%, 5%, and 10% levels, respectively; z-statistics in (). FGLS = Feasible Generalized Least Squares.

Source: Authors’ Computations.

**Table 5:** PCSE Results for the Regions (Dep. Variable: lnEMS).

*Variables*	*Central Africa*		*East Africa*		*North Africa*		*South Africa*		*West Africa*	
	[15]	[16]	[17]	[18]	[19]	[20]	[21]	[22]	[23]	[24]
ln*URBAN*	-2.120***	-2.209**	0.689***	0.568***	1.758***	1.377***	-0.550*	-0.583*	0.838***	0.256
	(-2.745)	(-2.294)	(2.940)	(2.597)	(4.067)	(3.462)	(-1.903)	(-1.875)	(4.830)	(0.969)
ln*ENERGY*	-0.00264	-0.386	1.483***	5.467***	0.560***	3.068***	1.652***	2.194	0.578***	4.441***
	(-0.0152)	(-0.187)	(5.625)	(4.694)	(5.671)	(3.442)	(9.119)	(1.248)	(5.972)	(3.025)
ln*INCOME*	1.871***	1.640	0.610***	3.656***	0.215***	2.453***	0.462	0.991	0.312***	3.863***
	(11.74)	(1.428)	(7.150)	(4.330)	(3.068)	(2.890)	(1.464)	(0.571)	(2.772)	(2.843)
lnENERGY*INCOME		0.0432		-0.486***		-0.288***		-0.0650		-0.559***
		(0.193)		(-3.627)		(-2.730)		(-0.311)		(-2.613)
*Net Effects*		0.00		2.15%		0.78%		0.00		0.59%
*CONSTANT*	0.000	0.000	0.000	0.000	0.000	0.000	-10.278***	-4.721	0.000	0.000
	(.)	(.)	(.)	(.)	(.)	(.)	(-8.01)	(-0.33)	(.)	(.)
No. of Obs./Groups	125/5	125/5	173/7	173/7	141/6	141/6	49/2	49/2	165/7	165/7
Wald Stat	1559.96***	1685.26***	1626.36***	2658.18***	2577.99***	5699.08***	881.07***	929.42***	1059.14***	3863.75***
R-Squared	0.852	0.850	0.887	0.886	0.849	0.931	0.938	0.937	0.769	0.930
Time Dummies	Yes	Yes	Yes	Yes	Yes	Yes	Yes	Yes	Yes	Yes

Note: ***, **, and * represent statistical significance at the 1%, 5%, and 10% levels, respectively; z-statistics in (); PCSE = Panel Corrected Standard Errors.

Source: Authors’ Computations.

Investigating the moderating role of income on energy use from the FGLS results, only East Africa (-0.455) and West Africa (-0.540) have statistically significant coefficients at the 1% and 5% levels, respectively. Taking a cue from previous equivalence, income moderates the devastating effect of energy on carbon emissions in East and West Africa. From the PCSE results, the coefficient of the interaction term is statistically significant for East Africa (-0.486), North Africa (-0.286), and West Africa (-0.559). These indicate that income reduces the worsening impact of energy on carbons emissions in East, North, and West Africa such that the net effects in those regions becomes 2.10%, 1.42%, and 0.56%, respectively. Analogous computations for the PCSE model are 2.15%, 0.78%, and 0.59%, respectively. As previously explained, adjustments to using more environmentally friendly energy sources as a result of change in the income status may be responsible for these outcomes.

### 4.5 MM-QR results—full sample

Next, we examine the effect of energy usage and income on the distribution of carbon emissions across three quartiles (Q = 0.25, Q = 0.50, Q = 0.75). Table 6 display the estimates of the parameters in the location and scale functions for the linear and moderation models, together with z-statistics in parenthesis (estimated by bootstrap resampling). As previously explained, it is assumed that the scale function is linear so as to preserve the linearity of the quartiles and allow for comparison with the estimates obtained with the FGLS and PCSE techniques. The results from the linear model indicate that energy and income significantly increase emissions across the quartiles. These findings provide additional evidence that both variables are critical determinants of emissions. Furthermore, the nonlinear model reveals that the interaction of both variables has a reducing effect on emissions across the quartiles. To the end, the net effects of energy usage on emissions across the quartiles are computed as: 0.67%, 0.63%, and 0.58%, respectively. In other words, income significantly reduces the devastating impact of energy on emissions. Previous interpretations hold. These are significant incursions into the carbon emissions literature.

**Table 6:** Distributional Effects from MM-QR Technique, Full Sample (Dep. Variable: lnEMS).

*Variables*	Linear Model					Moderation Model				
	Location	Scale	Q = 0.25	Q = 0.50	Q = 0.75	Location	Scale	Q = 0.25	Q = 0.50	Q = 0.75
ln*URBAN*	0.235***	0.445***	-0.165**	0.209***	0.623***	0.219***	0.331***	-0.0866	0.187**	0.538***
	(3.379)	(10.70)	(-2.042)	(2.924)	(7.182)	(3.108)	(8.298)	(-1.074)	(2.559)	(6.494)
ln*ENERGY*	0.522***	0.0336	0.492***	0.520***	0.551***	2.111***	-0.170	2.269***	2.128***	1.947***
	(10.56)	(1.136)	(9.075)	(10.54)	(9.573)	(8.455)	(-1.203)	(8.138)	(8.512)	(6.763)
ln*INCOME*	0.964***	-0.155***	1.103***	0.973***	0.829***	2.234***	-0.202*	2.420***	2.253***	2.039***
	(25.76)	(-6.912)	(26.21)	(25.75)	(18.48)	(10.68)	(-1.705)	(10.36)	(10.75)	(8.450)
lnENERGY*INCOME						-0.201***	0.0166	-0.216***	-0.202***	-0.185***
						(-6.320)	(0.924)	(-6.097)	(-6.367)	(-5.045)
*Net Effects*						0.62%	0.00	0.67%	0.63%	0.58%
*CONSTANT*	-12.79	8.469	-20.41***	-13.29***	-5.421***	-25.73	7.344	-32.51***	-26.43***	-18.64***
			(-46.32)	(-41.31)	(-11.65)			(-86.58)	(-84.25)	(-50.44)
Year Dummies	Yes	Yes	Yes	Yes	Yes	Yes	Yes	Yes	Yes	Yes
Observations	653	653	653	653	653	653	653	653	653	653

Note: ***, **, and * represent statistical significance at the 1%, 5%, and 10% levels, respectively; z-statistics in (); MM-QR = Method of moments quantile regression.

Source: Authors’ Computations.

## 5 Conclusion

In contributing towards the actualization of the Sustainable Development Goal (SDG) 13 which is to *“take urgent action to combat climate change and its impacts”* and with carbon emissions as the principal driver of climate change, this paper investigates the role of per capita income in moderating the impact of energy use on carbon emissions. It further enhances the carbon emissions discourse using data on 28 selected African countries covering 1990 to 2019 by presenting empirical findings which fill a gap in the literature. This investigation takes a new position and highlights results on whether energy usage and per capita income engender carbon emissions and whether their interaction escalates or diminishes the net effect of energy usage on carbon emissions. Conclusions reveal, *inter alia*, that though both variables exert emissions-increasing properties their interaction causes emissions to lessen. For the aggregated sample and across all model specifications and empirical techniques, we find that the net effect of energy reduces after accounting for per capita income. In essence, income has a strong influence on carbon emissions via energy usage. These are significant contributions to the emissions which provide the justification for engaging this study. Our findings provoke further questions on whether developmental trade-offs are required that support economic growth without increasing carbon emissions and escalating climate risks? We leave this open for more constructive discussions on the issue of carbon emissions and its environmental effects.

## Supporting information

S1 Appendix (DOCX)

S1 Data (XLSX)
