# Supplementary material for: Energy use and the role of per capita income on carbon emissions in African countries
Source: PLoS One. 2021 Nov 4;16(11):e0259488. doi: 10.1371/journal.pone.0259488 (PMC8568119; doi:10.1371/journal.pone.0259488)
Supplement: S1 Appendix — (DOCX) [file pone.0259488.s001.docx]

**Appendix**

| **Table 1A List of Countries and Respective Statistics** | | | | | | |
| --- | --- | --- | --- | --- | --- | --- |
| S/No. | COUNTRY | REGION | EMS | ENERGY | INCOME | URBAN |
| 1 | Algeria | North Africa | 3.182992 | 975.9451 | 4042.195 | 63.16177 |
| 2 | Angola | Central Africa | 0.867253 | 478.3472 | 2862.972 | 53.7752 |
| 3 | Benin | West Africa | 0.350553 | 347.6878 | 998.248 | 40.7654 |
| 4 | Botswana | Southern Africa | 2.264291 | 1053.187 | 5937.981 | 57.0628 |
| 5 | Cameroon | Central Africa | 0.23959 | 381.9213 | 1258.218 | 48.2675 |
| 6 | Congo, Dem. Rep. | Central Africa | 0.042058 | 318.0873 | 379.3493 | 37.4609 |
| 7 | Congo, Rep. | Central Africa | 0.476321 | 338.8831 | 2648.99 | 60.76773 |
| 8 | Cote d'Ivoire | West Africa | 0.40623 | 453.3822 | 1356.034 | 45.10327 |
| 9 | Egypt, Arab Rep. | North Africa | 2.020147 | 713.0716 | 2203.842 | 42.92467 |
| 10 | Ethiopia | East Africa | 0.07319 | 482.4154 | 298.4536 | 16.1885 |
| 11 | Gabon | Central Africa | 3.594517 | 1840.037 | 10024.71 | 81.29363 |
| 12 | Ghana | West Africa | 0.385359 | 331.4724 | 1190.63 | 46.83103 |
| 13 | Kenya | East Africa | 0.275935 | 444.9151 | 932.9828 | 21.7046 |
| 14 | Libya | North Africa | 8.728623 | 2862.851 | 8930.591 | 77.39877 |
| 15 | Mauritius | East Africa | 2.443574 | 868.1231 | 6821.095 | 42.2037 |
| 16 | Morocco | North Africa | 1.383399 | 431.0688 | 2447.909 | 55.5903 |
| 17 | Mozambique | East Africa | 0.113081 | 418.5104 | 385.8379 | 30.43913 |
| 18 | Namibia | Southern Africa | 1.14259 | 640.5587 | 4741.083 | 37.40353 |
| 19 | Niger | West Africa | 0.069406 | 131.0961 | 475.973 | 16.0869 |
| 20 | Nigeria | West Africa | 0.575814 | 729.6253 | 1878.715 | 39.3368 |
| 21 | Senegal | West Africa | 0.486028 | 249.5381 | 1213.41 | 42.27973 |
| 22 | South Africa | Southern Africa | 8.748569 | 2574.792 | 6616.934 |  |
| 23 | Sudan | North Africa | 0.232261 | 384.6562 | 1268.63 | 32.72337 |
| 24 | Tanzania | East Africa | 0.130909 | 421.8033 | 660.1985 | 25.44023 |
| 25 | Togo | West Africa | 0.282582 | 413.0291 | 567.6178 | 35.1095 |
| 26 | Tunisia | North Africa | 2.155557 | 780.2005 | 3413.246 | 64.61183 |
| 27 | Zambia | East Africa | 0.228519 | 625.408 | 1235.409 | 38.57387 |
| 28 | Zimbabwe | East Africa | 1.036033 | 827.9155 | 1222.113 | 32.65757 |
| Note: EMS = Carbons emissions per capita; ENERGY = non-renewable energy per capita; INCOME = GDP per capita; URBAN = Urban population (% of total population). Source: Authors' Computations from World Bank (2020) | | | | | | |
